# Transcriptomic evidence of an IFN-gamma-associated inflammatory state in active visceral leishmaniasis: an exploratory comparison with pediatric HLH

**DOI:** 10.3389/fcimb.2026.1924856

**Published:** 2026-10-01

**Authors:** Taoli Suo, Jing Zhang, Yanli Chen, Hongyu Shi, Haiyan Lu

**Affiliations:** Department of Hematology, Children’s Hospital of Shanxi, Women Health Center of Shanxi, Taiyuan, Shanxi, China

**Keywords:** CXCL10, CXCL9, hemophagocytic lymphohistiocytosis, IFN-gamma, single-cell transcriptome, STAT1, visceral leishmaniasis

## Abstract

**Background:**

Visceral leishmaniasis (VL) can progress to secondary hemophagocytic lymphohistiocytosis (HLH), but transcriptomic similarities between active VL and pediatric HLH remain uncertain.

**Methods:**

We integrated VL whole-blood expression data (GSE125992), sorted CD68-positive monocyte bulk-expression data from pediatric HLH and severe sepsis (GSE150707), and supportive Leishmania-infected mouse spleen single-cell data (GSE240385). Differential expression, pathway enrichment, an exploratory 132-gene HLH-versus-sepsis-derived ssGSEA score, FARDEEP-LM22 deconvolution, signature-excluded WGCNA, CellChat, and a refitted three-gene classifier were evaluated.

**Results:**

Active VL showed IFN-gamma-associated expression changes, including higher CXCL9 and CXCL10. The exploratory 132-gene score was higher in ActiveCase than EHC samples (Mann-Whitney P = 0.000637). Across 22 deconvolved cell types, only M1 macrophages showed an FDR-supported positive correlation with this score (rho = 0.787, BH q = 0.000503). After all 132 score genes were excluded before network construction, a 124-gene magenta module remained associated with the score (rho = 0.837, BH q = 4.29 × 10^-5). A refitted IFNG/GBP5/MX2 classifier achieved a same-study validation AUC of 0.917 (bootstrap 95% CI 0.733-1.000), but the validation set contained only 16 samples.

**Conclusion:**

These public datasets support an IFN-gamma-associated inflammatory state in active VL and exploratory cross-compartment convergence with pediatric HLH. The 132-gene score, deconvolution, network, and classifier results are hypothesis-generating and do not establish HLH specificity, cellular causality, independent external validity, or clinical HLH prediction.

## Introduction

Hemophagocytic lymphohistiocytosis (HLH) is a rapidly progressive hyperinflammatory syndrome with extremely high mortality, and its core pathological basis is the persistent uncontrolled amplification of the immune system and the formation of a cytokine storm (Griffin et al., 2020). Recent studies suggest that aberrant activation of monocytes and macrophages, sustained release of IFN-γ by CD8^+^ T cells and natural killer (NK) cells, and excessive activation of the STAT1-centered inflammatory transcriptional program collectively drive the pathological progression of HLH (Griffin et al., 2020; Cron et al., 2023). In particular, the IFN-γ-mediated chemokine amplification axis, such as CXCL9/CXCL10, is considered a key bridge linking cytotoxic lymphocyte activity to myeloid inflammatory amplification and is closely associated with disease severity (Lin et al., 2021). Therefore, systematic dissection of the IFN-related inflammatory network, myeloid inflammatory programs, and their quantifiable phenotypes remains a core scientific issue in pediatric HLH research.

Infection-associated secondary HLH accounts for a significant proportion in the pediatric population, indicating that pathogen-induced immune dysregulation is relevant to HLH development. Different pathogens can induce inflammatory myeloid activation through persistent antigen stimulation, innate immune-receptor signaling, and cytokine-network remodeling. At the peripheral-blood transcriptome level, enhanced IFN-related responses, chemokine-mediated immune-cell recruitment, and myeloid inflammatory programs have been described in hyperinflammatory syndromes (Dunmire et al., 2014). However, infection-associated inflammation and features observed in HLH are not interchangeable; transcriptomic overlap can indicate shared inflammatory programs but cannot identify an uncontrolled HLH state without clinical phenotyping.

Visceral leishmaniasis (VL), caused by infection with a protozoan of the genus Leishmania, is characterized by long-term involvement of the monocyte-macrophage system and complex immune activation and suppression. A subset of pediatric patients with VL can develop secondary HLH, with increased clinical risk (Guo et al., 2020). This clinical association motivates testing whether active VL exhibits transcriptional features that overlap with inflammatory programs observed in pediatric HLH, while recognizing that such overlap cannot determine progression to HLH.

However, current research on VL-associated HLH remains fragmented. Molecular datasets from clinically characterized patients with VL with and without HLH are lacking, and existing studies have not established whether VL whole blood and pediatric HLH monocytes share a reproducible transcriptional state after accounting for compartment and cellular-composition differences. An exploratory score can summarize cross-dataset similarity, but it cannot substitute for clinical HLH phenotyping or demonstrate a shared mechanism.

Accordingly, this study combined VL host-transcriptome discovery, exploratory features from sorted CD68-positive monocytes in pediatric HLH, supportive mouse single-cell contextualization, signature-excluded co-expression analysis, and classifier development with same-study experiment-level validation. An exploratory 132-gene HLH-versus-sepsis-derived score was calculated by ssGSEA (Hänzelmann et al., 2013). FARDEEP-LM22 deconvolution (Hao et al., 2019), WGCNA (Langfelder and Horvath, 2008), and CellChat (Jin et al., 2021) were used as association-level analyses. The resulting framework is exploratory and requires validation in clinically characterized VL cohorts.

## Materials and methods

### Public data download and transcriptome data acquisition

To characterize inflammatory transcriptomic features in VL and pediatric HLH, public datasets GSE125992, GSE150707, and GSE240385 were downloaded from the GEO database (https://www.ncbi.nlm.nih.gov/geo/). GSE125992 comprises peripheral-blood microarray profiles from active VL and endemic healthy controls (Fakiola et al., 2019). GSE150707 is an Affymetrix Human Transcriptome Array 2.0 bulk-expression dataset generated from sorted CD68-positive monocytes, including pediatric HLH samples (n = 12) and severe-sepsis samples (n = 8), and was used as an exploratory comparator cohort (Lin et al., 2021). GSE240385 is a mouse spleen single-cell transcriptome dataset from Leishmania infection and was used only for supportive cell-origin and CellChat contextualization (Karagiannis et al., 2023). GSE125991 served as the same-study experiment-level validation cohort for a newly refitted and fully specified classifier; this analysis was not treated as independent external validation because participant-level overlap could not be excluded from public metadata (Fakiola et al., 2019). Available cohort metadata, sample sizes, platforms, and unavailable clinical fields are summarized in **Table 1**; **Supplementary Table 1**.

**Table 1:** Clinical, demographic, and experimental-design metadata for the four GEO datasets.

Dataset	Species/source	Platform/assay	Deposited/analyzed	Age	Sex	Diagnosis/grouping	Disease phase	Comorbidities	Treatment status	Analytical role/limitation
GSE125992	Homo sapiens; whole blood; Bihar, India	GPL10558; Illumina HumanHT-12 v4 expression array	48 deposited; all exp2 analyses used 21 samples (ActiveCase n = 11; EHC n = 10)	Active VL: 21.6 +/- 9.4 y; all healthy controls (n=25): 24.8 +/- 17.6 y; EHC-only value NR	Active VL M:F 68%:32%; all healthy controls M:F 36%:64%; EHC-only value NR	Active VL: fever >2 weeks, splenomegaly, rK39 seropositivity and/or amastigotes in splenic aspirate; EHC: DAT <=1:1600 and modified Quantiferon-negative	ActiveCase sampled before treatment; EHC without clinical VL	NR	Baseline ActiveCase samples were pretreatment; source exp2 cohort subsequently received single-dose liposomal amphotericin B (10 mg/kg)	VL discovery, pathway enrichment, scoring, FARDEEP-LM22 deconvolution, signature-excluded WGCNA, and classifier training used all 21 eligible exp2 samples; whole-blood composition and incomplete EHC-specific metadata limit interpretation
GSE125991	Homo sapiens; whole blood; Bihar, India	GPL10558; Illumina HumanHT-12 v4 expression array	36 deposited; exp1 ActiveCase n = 10 and EHC n = 6 used for same-study experiment-level validation	Active VL: 31.0 +/- 12.6 y; all healthy controls (n=16): 40.9 +/- 17.8 y; EHC-only value NR	Active VL M:F 78%:22%; all healthy controls M:F 44%:56%; EHC-only value NR	Same clinical definitions as GSE125992; EHC was DAT-low and modified Quantiferon-negative	ActiveCase sampled before treatment; EHC without clinical VL	NR	Baseline ActiveCase samples were pretreatment; source exp1 cohort subsequently received 15 doses of non-liposomal amphotericin B	Same-study experiment-level validation of the refitted frozen IFNG/GBP5/MX2 classifier; belongs to GSE125993 with GSE125992 and is not an independent external cohort
GSE150707	Homo sapiens; peripheral-blood sorted CD68-positive monocytes	GPL17586; Affymetrix Human Transcriptome Array 2.0 bulk expression	42 deposited; CD68-positive subset: active HLH n=12 and severe sepsis n=8 analyzed	Pediatric cohort; exact age distribution for the 20-sample transcriptomic subset NR	NR for the transcriptomic subset	Active HLH by HLH-2004 criteria; severe sepsis by the 2005 International Pediatric Sepsis Consensus definitions	Newly diagnosed/active HLH or severe sepsis at blood collection	Participant-level triggers and comorbidities were not linked to the transcriptomic samples	Study excluded substantial pre-draw immunosuppression; sepsis/SIRS subjects received no immunosuppression before collection	Pediatric HLH disease anchor; sorted-cell compartment differs from VL whole blood and available clinical metadata are incomplete
GSE240385	Mus musculus (Black/6); spleen and bone marrow	GPL24247; Illumina NovaSeq 6000; 10x Genomics 3-prime scRNA-seq	4 deposited (spleen n=2, bone marrow n=2); spleen n=2 used	9–10 weeks	Female	Mice infected with 1 x 10^7 Leishmania donovani parasites; no uninfected control samples in the accession	Chronic infection; tissue harvested 72 days post-infection	Not applicable/NR	No therapeutic intervention reported	Cross-species single-cell contextualization only; n=2 spleen samples and absence of uninfected controls preclude human validation

Values describe the analytic groups used in this study. For GSE125991 and GSE125992, demographic values for the broader source-study healthy-control groups were not separable for the EHC-only subsets and are labeled accordingly. GSE150707 age, sex, trigger/comorbidity and treatment details were not linked to the 20 CD68-positive transcriptomic samples. NR, not reported for the analytic subset; DAT, direct agglutination test; EHC, endemic healthy control; HLH, hemophagocytic lymphohistiocytosis; VL, visceral leishmaniasis. Sources: GEO accession records and source studies (Fakiola et al., 2019; Lin et al., 2021; Karagiannis et al., 2023).

Datasets were eligible when they provided accessible raw or processed expression data, unambiguous sample annotations, and clinical or biological relevance to a prespecified analytical role. Human case-control inference additionally required an explicit comparator group; datasets without an appropriate comparator were restricted to contextual analyses. The cohort-specific eligibility decisions and analytical roles are detailed in **Supplementary Table 1**.

### Microarray data preprocessing and normalization analysis

According to the GEO records, these values were generated after background correction, quantile normalization, log2 transformation, and removal of non-expressed or unreliable probes; no additional between-array normalization was applied for the classifier analysis. For GSE150707, deposited log2-scale series-matrix expression values were imported, and no additional between-array normalization was applied. Probe or transcript-cluster IDs were mapped to gene symbols using the corresponding GPL annotation files. When multiple features corresponded to the same gene, the feature with the highest average expression was retained. No additional feature-level missingness or low-abundance filter was applied in the present reanalysis. Principal component analysis was performed on the processed expression matrices to inspect sample-level structure.

### Quality control and clustering analysis of single-cell transcriptome data

To identify the cellular context of selected IFN-associated inflammatory genes, single-cell transcriptome data from GSE240385 were analyzed using the Seurat package (version 4.3.0) (Stuart et al., 2019). The expression matrix was imported, Seurat objects were constructed, and standard quality-control metrics, including nFeature_RNA, nCount_RNA, and mitochondrial-gene percentage, were calculated. Cells were normalized using the LogNormalize method, highly variable genes were identified, data were scaled, and PCA, neighbor finding, clustering, and UMAP visualization were performed. GSE150707 was not analyzed as a single-cell dataset.

### Differential expression analysis

To identify inflammatory genes in active VL and pediatric HLH, differential-expression analysis was performed separately on GSE125992 and GSE150707. All 21 eligible GSE125992 exp2 samples (ActiveCase n = 11; EHC n = 10) were analyzed; no sample was excluded to equalize group sizes. Array-level comparisons used limma linear models (Ritchie et al., 2015) followed by empirical Bayes moderation with trend and robust estimation and Benjamini-Hochberg correction. The GSE125992 gene-level values used for downstream visualization were taken from the collapsed expression matrix and the same all-sample limma model. GSE150707 was analyzed as a sample-level bulk-expression cohort comparing pediatric HLH with severe sepsis. No GSE150707 gene met both |log2FC| > 1 and global BH q < 0.05. An explicitly exploratory list was therefore defined by |log2FC| > 1 and nominal P < 0.05 (212 upregulated and 62 downregulated genes). The GSE125992 VL-upregulated set required log2FC > 1 and BH q < 0.05 (n = 268); its intersection with the exploratory GSE150707 upregulated list contained 14 genes.

### Gene ontology, Kyoto encyclopedia of genes and genomes, and reactome functional enrichment analysis

To characterize pathways associated with the differentially expressed genes, Gene Ontology biological process (GO-BP) (Gene Ontology Consortium, 2021), Kyoto Encyclopedia of Genes and Genomes (KEGG) (Kanehisa et al., 2023), and Reactome (Gillespie et al., 2022) over-representation analyses were performed using hypergeometric tests and the relevant annotated-gene background. Gene identifiers were mapped with org.Hs.eg.db, KEGG pathway definitions were obtained through the KEGG REST resource, and Reactome pathway definitions were read from the Reactome GMT resource and tested by hypergeometric analysis using the corresponding annotated-gene background. For the GSE125992 and GSE150707 cohort-specific lists, gene sets containing 10–500 annotated background genes were tested; for the smaller 14-gene cross-cohort intersection, the lower gene-set-size bound was 5. Pathway-level P values were adjusted by the Benjamini-Hochberg method, and BH q < 0.05 was used for pathway-level inference. Results were visualized using ggplot2.

### Gene set enrichment analysis

To evaluate prespecified interferon- and cytokine-related pathways without relying only on a differential-expression cutoff, preranked gene set enrichment analysis (Subramanian et al., 2005) was performed on genes ordered by the limma log2 fold change. Reactome and KEGG gene sets containing 10–500 represented genes were tested with 1,000 permutations. Normalized enrichment scores (NES), nominal P values, and Benjamini-Hochberg q values were reported. BH q < 0.05 was used for inferential significance; the prespecified interferon gamma signaling curve was retained for descriptive review even when it did not meet this threshold.

### Calculation of the exploratory 132-gene cross-compartment score

An exploratory HLH-versus-sepsis-derived score was defined from the 212 GSE150707 genes upregulated at log2FC > 1 and nominal P < 0.05. After platform mapping, 132 genes were represented in GSE125992. Rank-normalized ssGSEA normalized enrichment scores were calculated across all 21 GSE125992 exp2 samples (ActiveCase n = 11; EHC n = 10) and used in downstream group, correlation, deconvolution, and signature-excluded network analyses. The 132-gene score is an exploratory cross-compartment transcriptional measure and is not HLH-specific, diagnostic, or clinically validated. For a separate sensitivity analysis, official human MSigDB HALLMARK_HEME_METABOLISM (M5945) and HALLMARK_INFLAMMATORY_RESPONSE (M5932) gene sets were used (Liberzon et al., 2015), with HMOX1 excluded before scoring. Two-sided Spearman tests and 2,000 sample-level bootstrap resamples were used as described in the figure legend.

### Immune cell infiltration deconvolution analysis

To assess estimated immune-cell composition during active VL, all 21 GSE125992 exp2 samples (ActiveCase n = 11; EHC n = 10) were deconvolved using FARDEEP version 1.0.1 (Hao et al., 2019) with the bundled LM22 matrix (Newman et al., 2015). The fardeep function used 1,000 permutations, quantile normalization, non-negative coefficients, and an intercept (permn = 1000, QN = TRUE, nn = TRUE, intercept = TRUE). Relative fractions were normalized to sum to one; all 21 samples had deconvolution P < 0.05. Composition profiles and four group comparisons were summarized with Benjamini-Hochberg correction across those four tests. The exploratory 132-gene score was correlated with all 22 LM22 cell types using two-sided Spearman tests and Benjamini-Hochberg correction across the complete 22-test family. Resting mast-cell and resting dendritic-cell fractions were constant at zero and were retained conservatively with P = 1 and q = 1. Percentile 95% confidence intervals used 2,000 sample-level bootstrap resamples. Detailed group-comparison and correlation results are reported in **Supplementary Tables 2, 3**.

### Correlation analysis and co-expression network construction

Weighted Gene Co-expression Network Analysis was rerun *de novo* in all 21 GSE125992 exp2 samples after excluding all 132 score genes before variability filtering or network construction. The 5,000 remaining genes with the highest median absolute deviation were analyzed using a signed network, biweight midcorrelation, deepSplit = 2, minimum module size = 30, and merge cut height = 0.25. Candidate powers 1–20 did not reach signed scale-free topology R² >= 0.80; therefore, power 16 was used as the prespecified small-sample fallback. Module eigengenes were correlated by Spearman tests with ActiveCase status, the exploratory 132-gene score, M1 macrophage fractions, and CD8-positive T-cell fractions, with Benjamini-Hochberg correction across all 56 module-trait tests. The analysis used WGCNA version 1.74 with random seed 20260811.

### CellChat cell–cell communication analysis

To explore inferred ligand-receptor communication in the Leishmania-infected mouse spleen context, CellChat version 1.6.1 (Jin et al., 2021) was applied to GSE240385 using CellChatDB.mouse. Overexpressed genes and interactions were identified, communication probabilities were calculated, and aggregate networks were constructed. The analysis focused on IFNG-IFNGR and CXCL9/CXCL10-CXCR3 interactions among CD8-positive T cells, NK cells, T cells, macrophages, and monocytes. These transcript-based probabilities were interpreted as supportive associations and not as evidence of cytokine secretion or causal signaling.

### Active-VL inflammatory classifier construction and ROC analysis

To refit an active-VL inflammatory classifier, LASSO logistic regression was performed in GSE125992 (ActiveCase n = 11; EHC n = 10) using the glmnet package (version 4.1-10) (Friedman et al., 2010), with GSE125991 (ActiveCase n = 10; EHC n = 6) reserved for validation. IFNG, GBP5, and MX2 were treated as the prespecified feature set based on the reported model. Shared GPL10558 probes were selected using the highest mean expression in GSE125992 training samples only (IFNG, ILMN_2207291; GBP5, ILMN_2114568; MX2, ILMN_2231928). A fixed seed (20260811) and stratified ten-fold foldid were used with family = binomial, alpha = 1, type.measure = deviance, standardize = TRUE, and intercept = TRUE; cv.glmnet enforced grouped = FALSE because some folds contained fewer than three observations. The fixed folds selected lambda.min = 0.02878664, at which all three genes had nonzero coefficients. The frozen model was logit(p) = -18.7752806 + 0.7503904 x IFNG + 2.2059416 x GBP5 - 1.1916386 x MX2. The model was applied directly to GSE125991 without feature reselection, coefficient refitting, or threshold optimization, using a prespecified classification threshold of p = 0.5. AUCs and their confidence intervals were calculated with pROC (Robin et al., 2011) using 2,000 stratified bootstrap resamples. Exploratory calibration was summarized using the Brier score, calibration intercept and slope, three equal-frequency groups, and 2,000 stratified bootstrap resamples.

### Statistical analysis

All statistical analyses were performed in R or Python using the procedures described above. For ordinary two-group comparisons, the Wilcoxon rank-sum test was used when either group had Shapiro-Wilk P <= 0.05. When both groups had Shapiro-Wilk P > 0.05, Welch’s t-test was used if Levene’s P <= 0.05; otherwise, Student’s t-test was applied. Gene-level primary inference used limma empirical-Bayes models with transcriptome-wide Benjamini-Hochberg correction. The four displayed immune-cell group comparisons were adjusted as one family. Two-sided Spearman tests were used for score-gene, score-cell, and module-trait associations; score-cell correlations were corrected across 22 cell types and module-trait correlations across 56 tests. Bootstrap confidence intervals used 2,000 resamples unless otherwise stated. Detailed assumption checks and retained-source comparison statistics are reported in **Supplementary Table 2**.

## Results

### Differential expression and pathway-enrichment features in active visceral leishmaniasis

Differential-expression analysis of all 21 GSE125992 exp2 samples (ActiveCase n = 11; EHC n = 10) showed broad transcriptomic differences between groups. The heatmap displayed higher ActiveCase expression of CXCL9, GBP5, CXCL10, GBP1, and STAT1 and lower expression of CD163, HMOX1, S100A8, and S100A9 (**Figure 1A**). At log2FC > 1 and BH q < 0.05, 268 genes were upregulated, whereas 370 genes were downregulated at log2FC < -1 and BH q < 0.05 (**Figure 1B**). These findings establish extensive expression remodeling in active VL but do not by themselves identify a causal cellular mechanism.

**Figure 1:**
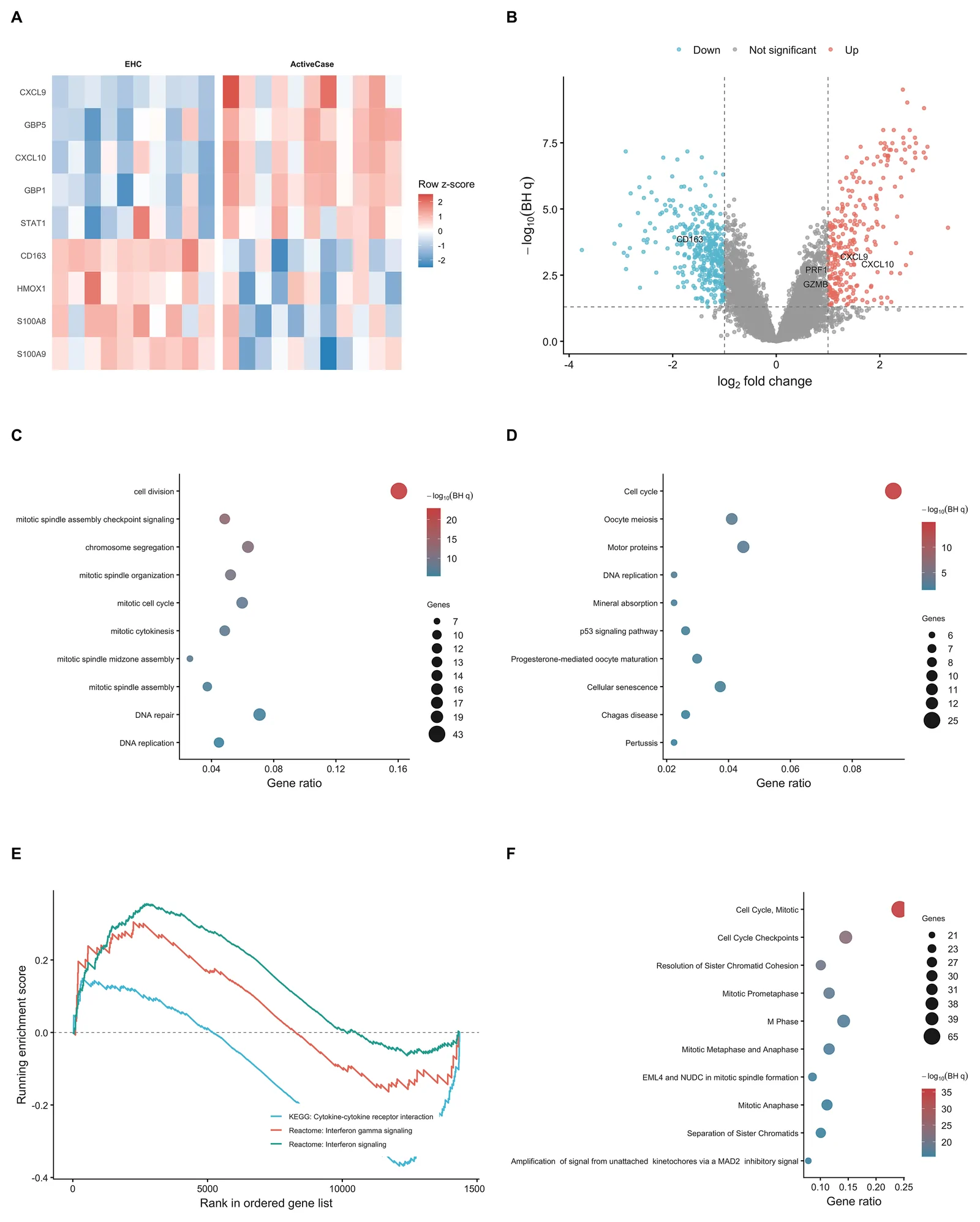
Differential expression and pathway-enrichment features in active visceral leishmaniasis. All panels use the 21 eligible GSE125992 exp2 samples (ActiveCase n = 11; EHC n = 10). **(A)** Z-score heatmap of CXCL9, GBP5, CXCL10, GBP1, STAT1, CD163, HMOX1, S100A8, and S100A9. **(B)** Limma volcano plot; 268 genes were upregulated and 370 were downregulated at |log2FC| > 1 and BH q < 0.05. **(C)** GO-BP over-representation analysis of the 268 strict upregulated genes, dominated by cell division, mitotic spindle, chromosome segregation, DNA repair, and DNA replication. **(D)** KEGG over-representation analysis, led by cell cycle and related pathways. **(E)** Prespecified preranked GSEA: Reactome interferon signaling, NES = 1.720 and BH q = 0.0140; Reactome interferon gamma signaling, NES = 1.238 and BH q = 0.209; KEGG cytokine-cytokine receptor interaction, NES = -1.622 and BH q = 0.0133. **(F)** Reactome over-representation analysis, dominated by mitotic cell-cycle and checkpoint pathways. ORA P values were BH-adjusted at the pathway level.

Over-representation analysis of the 268 strict ActiveCase-upregulated genes was dominated by cell-division, mitotic-spindle, chromosome-segregation, DNA-replication, and cell-cycle pathways (**Figures 1C, D, F**). In the prespecified GSEA panel, Reactome interferon signaling showed positive enrichment (NES = 1.720, BH q = 0.0140), whereas Reactome interferon gamma signaling did not meet the FDR threshold (NES = 1.238, BH q = 0.209). KEGG cytokine-cytokine receptor interaction showed negative rather than positive enrichment (NES = -1.622, BH q = 0.0133; **Figure 1E**). Thus, the all-sample analysis supports prominent proliferative or cell-cycle-associated expression together with a selective IFN-associated signal; these transcriptomic findings do not demonstrate macrophage hyperreactivity or a causal IFN-gamma mechanism.

### Exploratory differential-expression and pathway features in pediatric HLH CD68-positive monocytes

The sorted CD68-positive monocyte bulk-expression cohort GSE150707 was analyzed at the sample level. Principal component analysis described the structure of pediatric HLH (n = 12) and severe sepsis (n = 8) samples (**Figure 2A**). No gene met both |log2FC| > 1 and global BH q < 0.05. For transparent exploratory visualization, |log2FC| > 1 with nominal P < 0.05 identified 212 upregulated and 62 downregulated genes (**Figure 2B**), and the 30 exploratory upregulated genes with the smallest nominal P values were displayed in the heatmap (**Figure 2C**).

**Figure 2:**
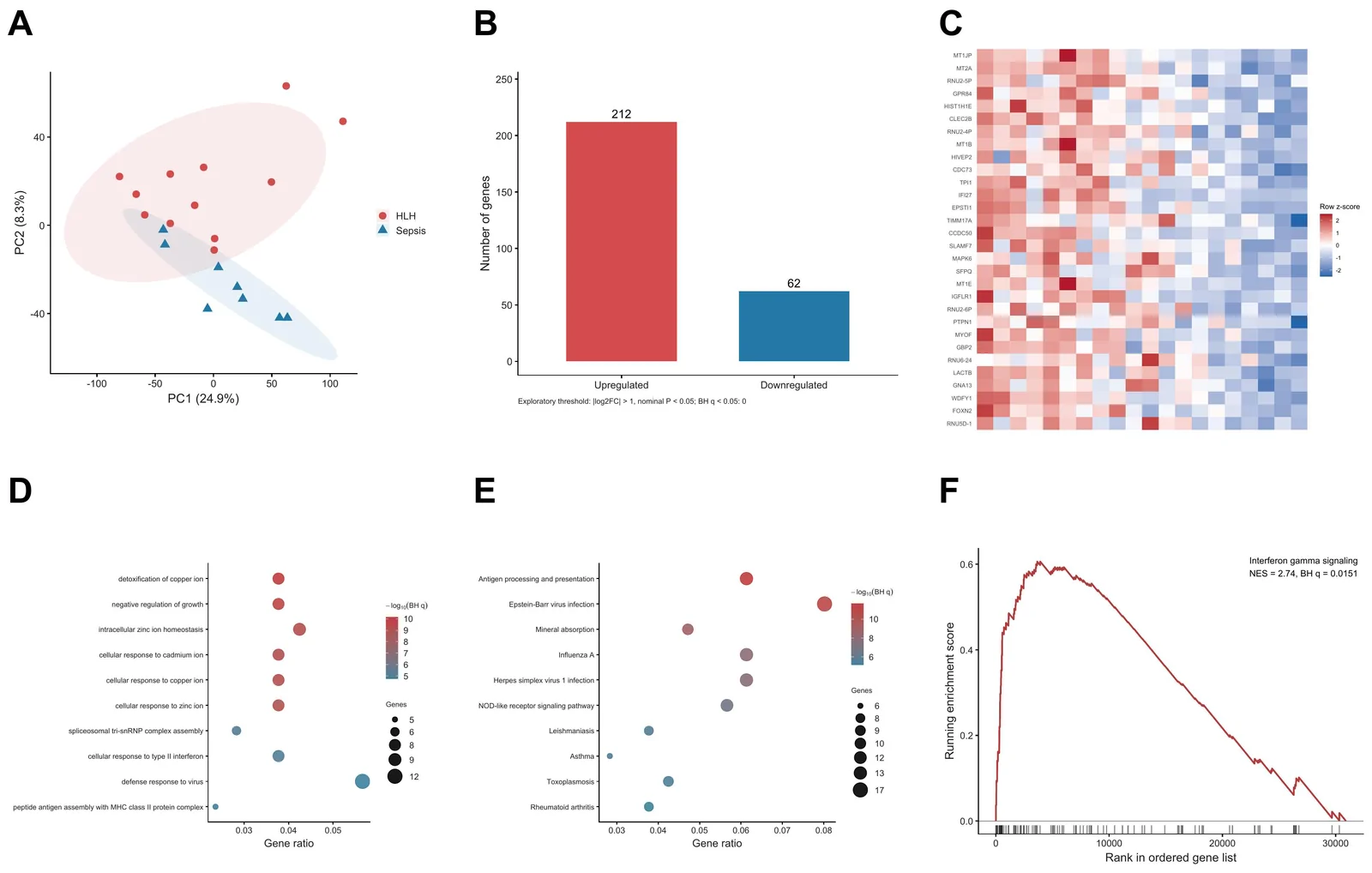
Sample-level reanalysis of the GSE150707 pediatric HLH and severe-sepsis bulk array dataset. **(A)** PCA of the GSE150707 Affymetrix Human Transcriptome Array 2.0 bulk-expression dataset comparing sorted CD68-positive monocytes from pediatric HLH (n = 12) and severe sepsis (n = 8). **(B)** Exploratory gene counts at |log2FC| > 1 and nominal P < 0.05: 212 upregulated and 62 downregulated; no gene met |log2FC| > 1 and global BH q < 0.05. **(C)** Heatmap of the 30 exploratory upregulated genes with the smallest nominal P values. **(D, E)** GO-BP **(D)** and KEGG **(E)** over-representation analyses of the exploratory 212-gene list using the array-annotated background and pathway-level BH correction. **(F)** Preranked Reactome GSEA across all annotated genes showing interferon gamma signaling (NES = 2.736, BH q = 0.0151).

GO-BP and KEGG over-representation analyses were applied to the exploratory 212-gene GSE150707 upregulated list using the array-annotated background; pathway-level P values were adjusted by the Benjamini-Hochberg method (**Figures 2D, E**). These analyses highlighted metal-ion response, type II interferon response, antiviral defense, antigen processing and presentation, and NOD-like receptor signaling, but remained conditional on the exploratory gene-selection threshold. A preranked Reactome GSEA across all annotated genes identified interferon gamma signaling (NES = 2.736, BH q = 0.0151; **Figure 2F**). After mapping to GSE125992, the same exploratory 212-gene list provided 132 represented genes for the exploratory score used in the dependent analyses; this score was not treated as an FDR-confirmed or HLH-specific signature.

### Exploratory 132-gene HLH-versus-sepsis-derived score in active VL

To evaluate cross-compartment transcriptional similarity, 132 of the 212 exploratory GSE150707 HLH-versus-sepsis upregulated genes were mapped to GSE125992 and summarized by rank-normalized ssGSEA. PCA of the full 21-sample expression matrix showed partial separation of ActiveCase (n = 11) and EHC (n = 10) samples (**Figure 3A**).

**Figure 3:**
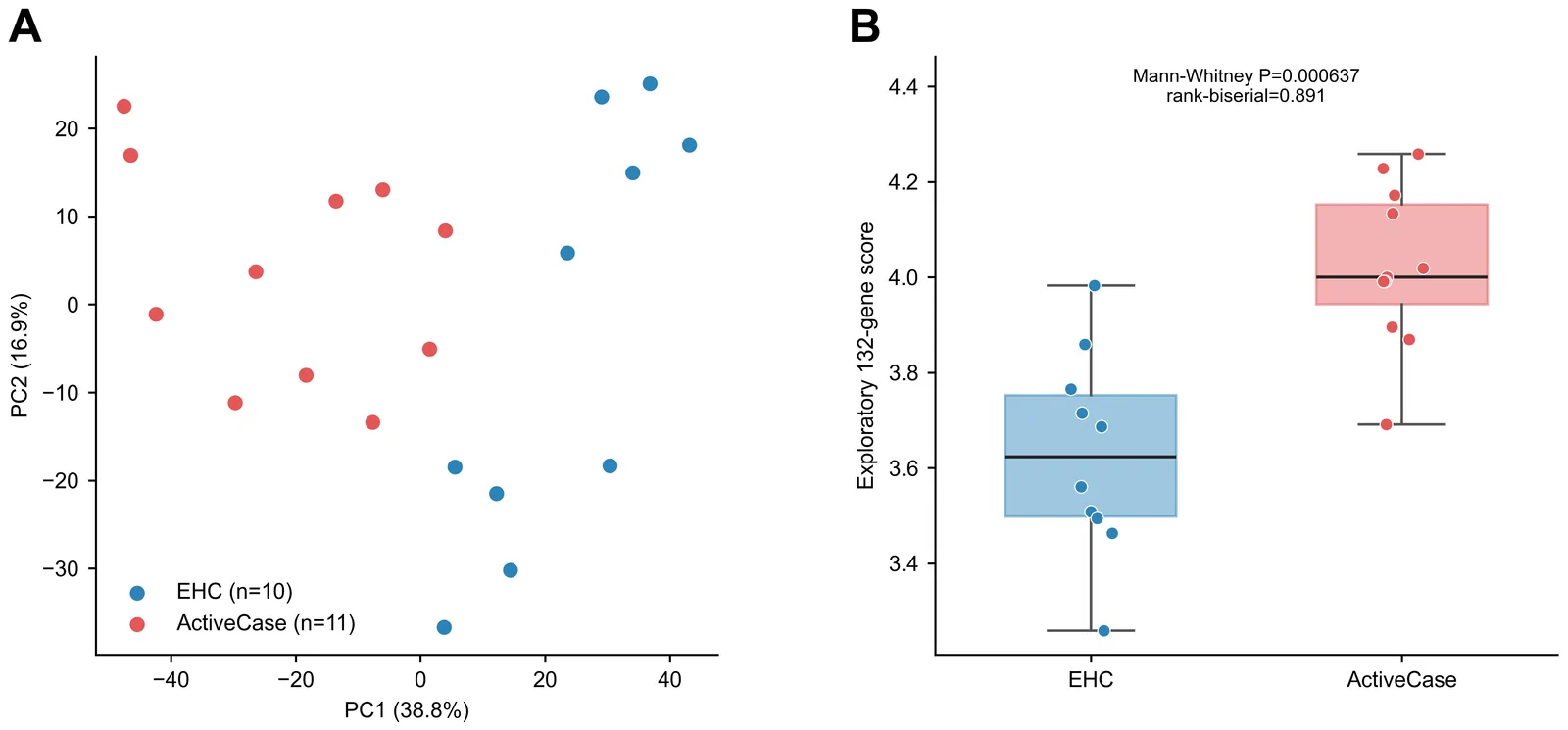
PCA structure and exploratory 132-gene HLH-versus-sepsis-derived score in GSE125992. **(A)** PCA of all 21 GSE125992 exp2 samples (ActiveCase n = 11; EHC n = 10), based on the 2,000 genes with the highest median absolute deviation; PC1 and PC2 explained 38.8% and 16.9% of variance, respectively. **(B)** Rank-normalized ssGSEA score based on 132 of 212 exploratory GSE150707 HLH-versus-sepsis upregulated genes that mapped to GSE125992. The score was higher in ActiveCase than EHC samples (Mann-Whitney P = 0.000637; rank-biserial correlation = 0.891; median difference = 0.377; 2,000-bootstrap 95% CI, 0.194-0.632). The score is exploratory and is not HLH-specific or diagnostic.

The exploratory 132-gene score was higher in ActiveCase than EHC samples (median difference = 0.377; Mann-Whitney P = 0.000637; rank-biserial correlation = 0.891; bootstrap 95% CI for the median difference, 0.194-0.632; **Figure 3B**). Because the score was derived from a nominal-P-selected pediatric HLH-versus-sepsis contrast in sorted monocytes and then projected into VL whole blood, this finding indicates cross-compartment transcriptional convergence only. It does not establish HLH specificity, clinical HLH, or a causal inflammatory mechanism.

### Exploratory cross-dataset intersection between active VL whole blood and pediatric HLH monocytes

The 14-gene intersection and the 132-gene exploratory score were retained as distinct analyses with different selection purposes.

Reactome, GO-BP, and KEGG enrichment analyses were performed on the 14-gene exploratory intersection using the 13,554 genes shared between the two annotated datasets as background and BH correction (**Figures 4B–D**). The intersection comprised 14 genes, with 254 genes unique to the strict VL-upregulated set and 198 unique to the exploratory HLH-upregulated set (**Figure 4A**). Reactome results included metallothionein or metal-response, GBP-mediated host-defense, and interferon gamma signaling terms; GO-BP results included metal-ion responses, type II interferon response, and antiviral defense. Mineral absorption (BH q = 2.54 × 10^-5) and NOD-like receptor signaling (BH q = 0.00157) met BH q < 0.05 in KEGG. Full gene-level membership and statistics are provided in **Supplementary Table 4**. These pathway-level results are exploratory and do not remove cross-platform, cellular-composition, or sample-compartment confounding.

**Figure 4:**
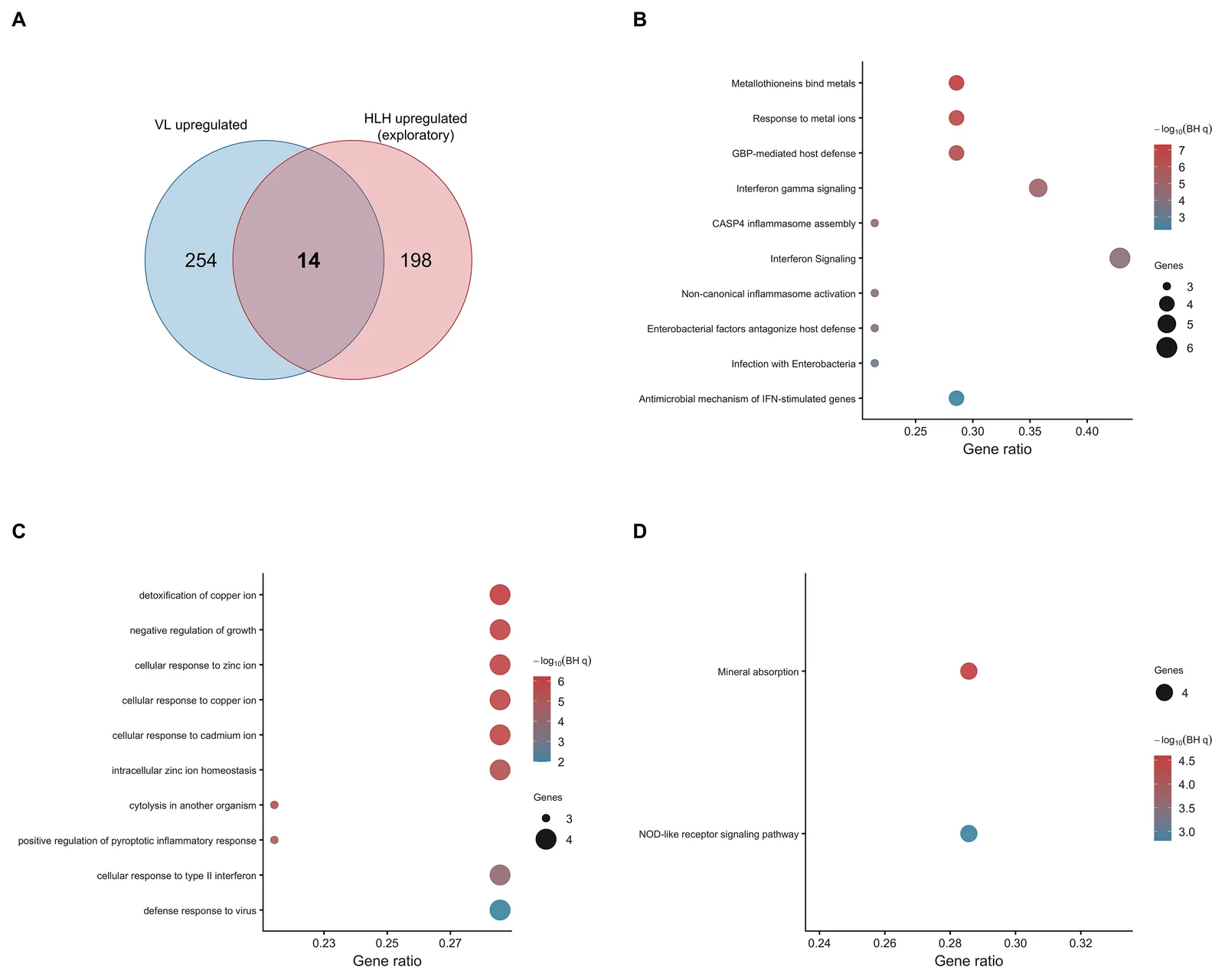
Reanalysis of shared upregulated inflammatory networks between visceral leishmaniasis and pediatric HLH. **(A)** Intersection of 268 strict GSE125992 VL-upregulated genes (log2FC > 1, BH q < 0.05) with 212 exploratory GSE150707 HLH-upregulated genes (log2FC > 1, nominal P < 0.05), yielding 14 overlapping, 254 VL-only, and 198 HLH-only genes after gene-ID harmonization. **(B–D)** Reactome **(B)**, GO-BP **(C)**, and KEGG **(D)** enrichment of the 14-gene exploratory intersection using 13,554 shared annotated genes as background and pathway-level BH correction. In KEGG, mineral absorption (BH q = 2.54 × 10^-5) and NOD-like receptor signaling (BH q = 0.00157) met BH q < 0.05.

### Expression and correlation patterns of IFN-gamma-associated genes across the two datasets

To examine IFN-gamma-axis expression across the two datasets, STAT1, CXCL9, and CXCL10 were evaluated separately in GSE125992 and GSE150707. In all 21 GSE125992 exp2 samples, STAT1 was modestly higher in ActiveCase than EHC (log2FC = 0.290, nominal P = 0.0298) but did not remain significant after transcriptome-wide BH correction (q = 0.116; **Figure 5A**). CXCL9 and CXCL10 were higher in ActiveCase and remained significant after BH correction (CXCL9: log2FC = 1.504, nominal P = 6.08 × 10^-5, BH q = 0.00129; **Figure 5B**; CXCL10: log2FC = 1.957, nominal P = 1.39 × 10^-4, BH q = 0.00244; **Figure 5C**). These within-cohort comparisons support IFN-gamma-associated transcription in active VL but do not establish equivalence with the sorted-monocyte HLH cohort.

**Figure 5:**
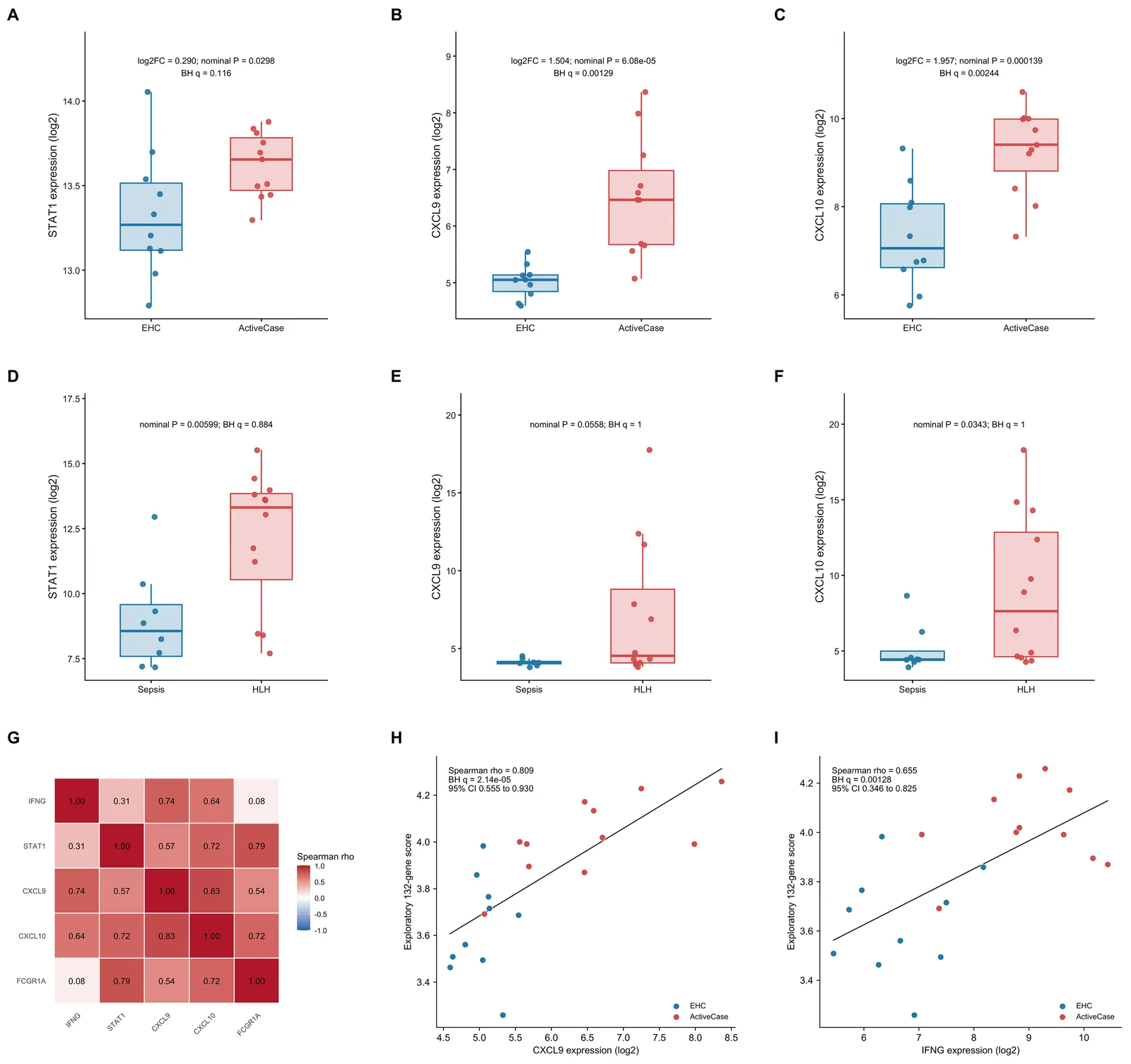
IFNG/STAT1/CXCL9-CXCL10 expression and exploratory 132-gene-score associations. **(A–C)** GSE125992 exp2 whole-blood expression across all 21 samples (ActiveCase n = 11; EHC n = 10): STAT1, log2FC = 0.290, nominal P = 0.0298, BH q = 0.116 **(A)**; CXCL9, log2FC = 1.504, nominal P = 6.08 × 10^-5, BH q = 0.00129 **(B)**; CXCL10, log2FC = 1.957, nominal P = 1.39 × 10^-4, BH q = 0.00244 **(C)**. **(D–F)** GSE150707 sorted CD68-positive monocytes from HLH (n = 12) and severe sepsis (n = 8): STAT1, nominal P = 0.00599, BH q = 0.884 **(D)**; CXCL9, nominal P = 0.0558, BH q = 1.000 **(E)**; CXCL10, nominal P = 0.0343, BH q = 1.000 **(F)**. **(G)** Spearman correlation matrix for IFNG, STAT1, CXCL9, CXCL10, and FCGR1A across all 21 GSE125992 samples, with BH correction across 10 pairs. **(H, I)** Correlations of the exploratory 132-gene score with CXCL9 (rho = 0.809, raw P = 8.96 × 10^-6, BH q = 2.14 × 10^-5; H) and IFNG (rho = 0.655, raw P = 0.00128, BH q = 0.00128; I).

The same genes were then examined in the GSE150707 CD68-positive monocyte bulk-expression cohort. For HLH versus severe sepsis, STAT1 showed log2FC = 3.15, nominal P = 0.00599, and BH q = 0.884 (**Figure 5D**); CXCL9 showed log2FC = 3.04, nominal P = 0.0558, and BH q = 1.000 (**Figure 5E**); and CXCL10 showed log2FC = 3.84, nominal P = 0.0343, and BH q = 1.000 (**Figure 5F**). Thus, none of these gene-level comparisons was significant after global BH correction. They were interpreted as exploratory comparator results in sorted monocytes, not as single-cell marker tests or FDR-confirmed replication.

Across all 21 GSE125992 exp2 samples, Spearman analysis assessed co-expression among IFNG, STAT1, CXCL9, CXCL10, and FCGR1A, with BH correction across the 10 pairwise correlations (**Figure 5G**). The exploratory 132-gene score correlated with CXCL9 (rho = 0.809, raw P = 8.96 × 10^-6, BH q = 2.14 × 10^-5; **Figure 5H**) and IFNG (rho = 0.655, raw P = 0.00128, BH q = 0.00128; **Figure 5I**). These within-cohort associations are consistent with IFN-gamma-associated transcription in active VL but do not establish an identical or causal mechanism across VL whole blood and pediatric HLH monocytes.

### Cytotoxic-effector-associated expression patterns in active VL

GZMB, PRF1, and FASLG expression was evaluated in all 21 GSE125992 exp2 samples (ActiveCase n = 11; EHC n = 10). Each gene was higher in ActiveCase in the limma analysis (GZMB: log2FC = 0.761, nominal P = 0.00152, BH q = 0.0141; **Figure 6A**; PRF1: log2FC = 0.775, nominal P = 2.72 × 10^-4, BH q = 0.00402; **Figure 6B**; FASLG: log2FC = 1.475, nominal P = 5.05 × 10^-6, BH q = 2.14 × 10^-4; **Figure 6C**). These expression differences indicate cytotoxic-effector-associated transcriptional activity but do not directly measure cytotoxic function.

**Figure 6:**
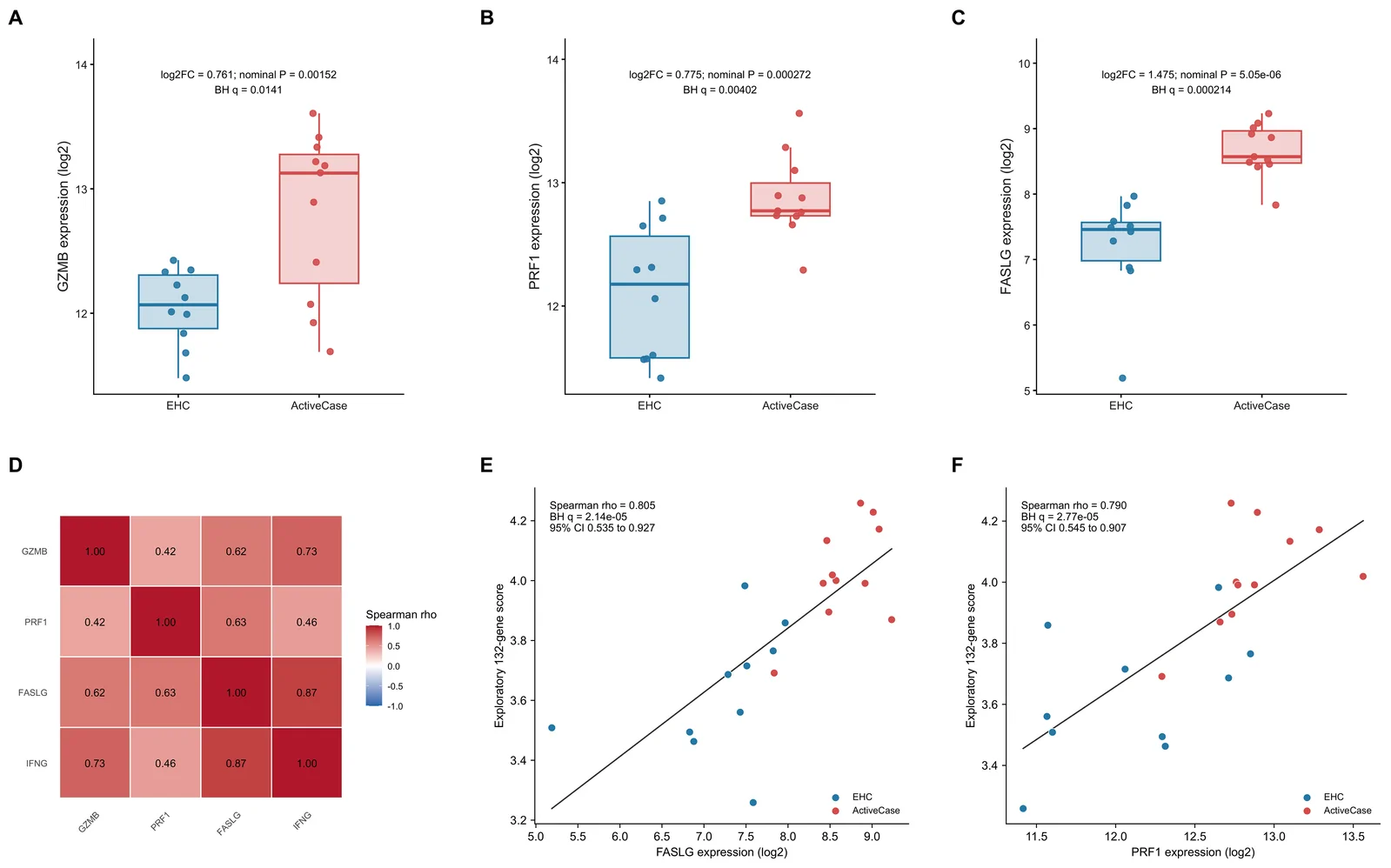
Cytotoxic-effector-associated expression and exploratory 132-gene-score correlations in active VL. **(A–C)** GSE125992 exp2 expression comparisons across all 21 samples (ActiveCase n = 11; EHC n = 10): GZMB, log2FC = 0.761, nominal P = 0.00152, BH q = 0.0141 **(A)**; PRF1, log2FC = 0.775, nominal P = 2.72 × 10^-4, BH q = 0.00402 **(B)**; FASLG, log2FC = 1.475, nominal P = 5.05 × 10^-6, BH q = 2.14 × 10^-4 **(C)**. **(D)** Spearman correlation matrix for GZMB, PRF1, FASLG, and IFNG across all 21 samples, with BH correction across six pairs; GZMB-PRF1 did not retain FDR support (rho = 0.420, BH q = 0.0584). **(E, F)** Correlations of the exploratory 132-gene score with FASLG (rho = 0.805, raw P = 1.07 × 10^-5, BH q = 2.14 × 10^-5; E) and PRF1 (rho = 0.790, raw P = 2.08 × 10^-5, BH q = 2.77 × 10^-5; F).

A Spearman correlation matrix summarized co-expression among GZMB, PRF1, FASLG, and IFNG across all 21 exp2 samples, with BH correction across six pairwise correlations (**Figure 6D**). Five pairs retained BH q < 0.05, whereas GZMB-PRF1 did not (rho = 0.420, BH q = 0.0584). Across the same 21 samples, the exploratory 132-gene score correlated with FASLG (rho = 0.805, raw P = 1.07 × 10^-5, BH q = 2.14 × 10^-5; **Figure 6E**) and PRF1 (rho = 0.790, raw P = 2.08 × 10^-5, BH q = 2.77 × 10^-5; **Figure 6F**). These associations are cross-sectional and do not demonstrate uncontrolled cytotoxic function or a pathogenic HLH mechanism.

### Immune-cell deconvolution with FARDEEP-LM22 and correlation analysis

FARDEEP-LM22 was applied to all 21 GSE125992 exp2 peripheral-blood samples (ActiveCase n = 11; EHC n = 10), and every sample had a deconvolution P < 0.05. **Figure 7A** displays the estimated relative fractions of all 22 LM22 immune-cell types and four prespecified group comparisons. After Benjamini-Hochberg correction across these four comparisons, ActiveCase samples had lower monocyte fractions (BH q = 0.0171) and higher M1 macrophage fractions (BH q = 0.0171) than EHC samples. CD8-positive T-cell (BH q = 0.188) and activated NK-cell (BH q = 0.0621) fractions did not meet the FDR threshold. Assumption checks, selected tests, effect estimates, and confidence intervals are reported in **Supplementary Table 2**.

**Figure 7:**
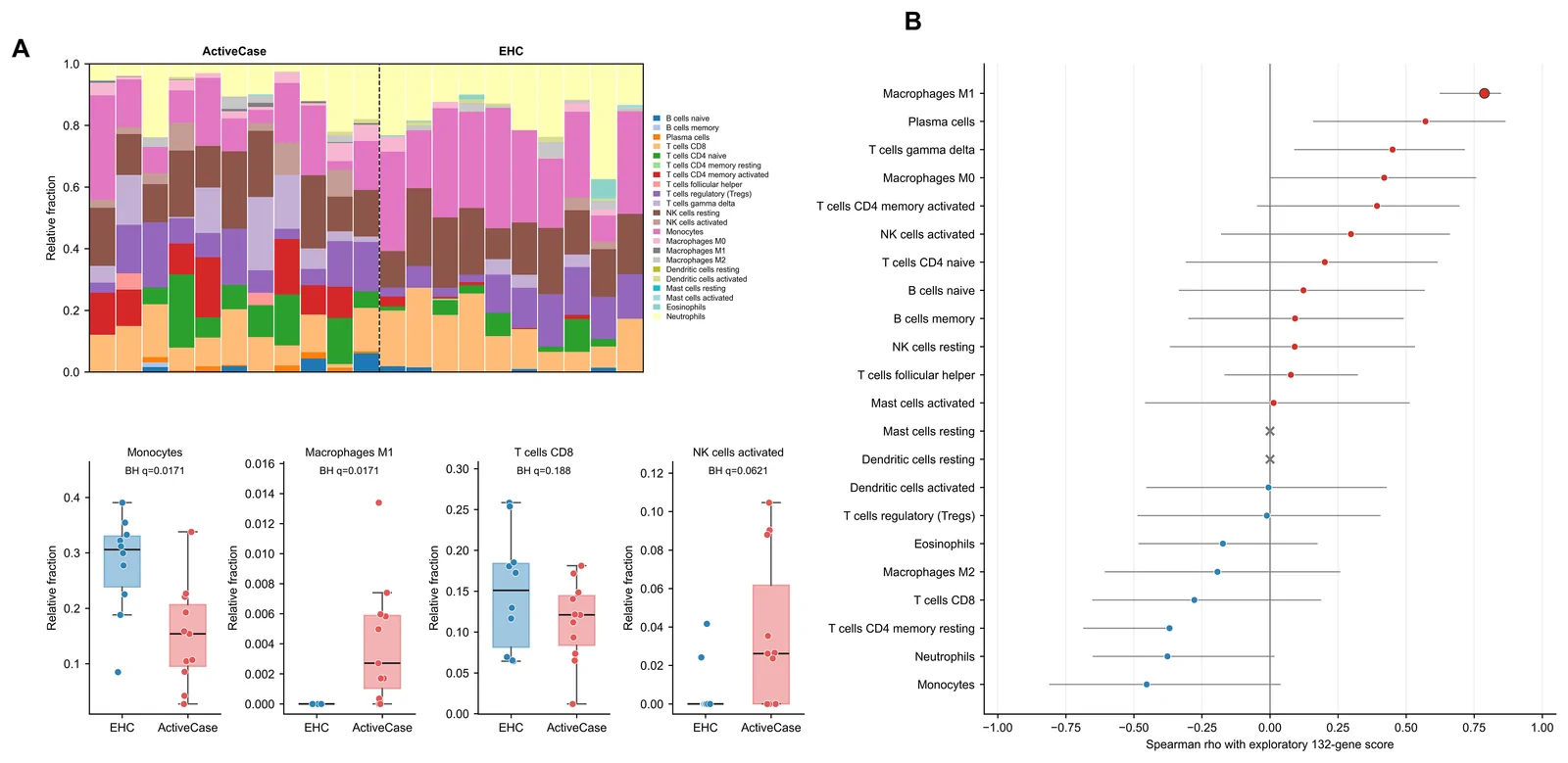
FARDEEP-LM22 immune-cell deconvolution and exploratory 132-gene-score associations in GSE125992. **(A)** FARDEEP version 1.0.1 with LM22 in all 21 GSE125992 exp2 samples (ActiveCase n = 11; EHC n = 10), using relative fractions, quantile normalization, and 1,000 permutations. For the four displayed group comparisons, BH q values were 0.0171 for monocytes, 0.0171 for M1 macrophages, 0.188 for CD8-positive T cells, and 0.0621 for activated NK cells. **(B)** Spearman correlations between the exploratory 132-gene score and all 22 LM22 cell fractions, with BH correction across all 22 tests. Only M1 macrophages were FDR-supported (rho = 0.787, raw P = 2.29 × 10^-5, BH q = 0.000503; 2,000-bootstrap 95% CI, 0.626-0.847). Resting mast cells and resting dendritic cells were constant at zero and not estimable; both were retained with P = 1 and q = 1. Full results are provided in **Supplementary Tables 2, 3**.

In the 21-sample analysis, only M1 macrophages retained FDR support across the complete 22-cell family (rho = 0.787, raw P = 2.29 × 10^-5^, BH q = 0.000503; bootstrap 95% CI, 0.626-0.847; **Figure 7B**; **Supplementary Table 3**). Associations for CD8-positive T cells, resting NK cells, and activated NK cells were not FDR-supported. Resting mast cells and resting dendritic cells had constant-zero fractions, so their correlations were not estimable and were conservatively retained with P = 1 and q = 1.

### Supportive mouse single-cell context and inferred CellChat communication

GSE240385 provided supportive mouse spleen single-cell context only. Fifteen major cell subsets were identified after quality control and clustering (**Figure 8A**), and the two infected spleen samples showed broadly similar cell-type composition profiles (**Figure 8B**). FeaturePlot distributions of Stat1, Cxcl9, Cxcl10, Isg15, and Gbp2 are shown in **Supplementary Figure 1A**, and the corresponding cell-type DotPlot summary is shown in **Supplementary Figure 1B**. Because the accession lacks an uninfected control and contains only two spleen samples, these observations do not constitute validation of the human whole-blood or sorted-monocyte findings.

**Figure 8:**
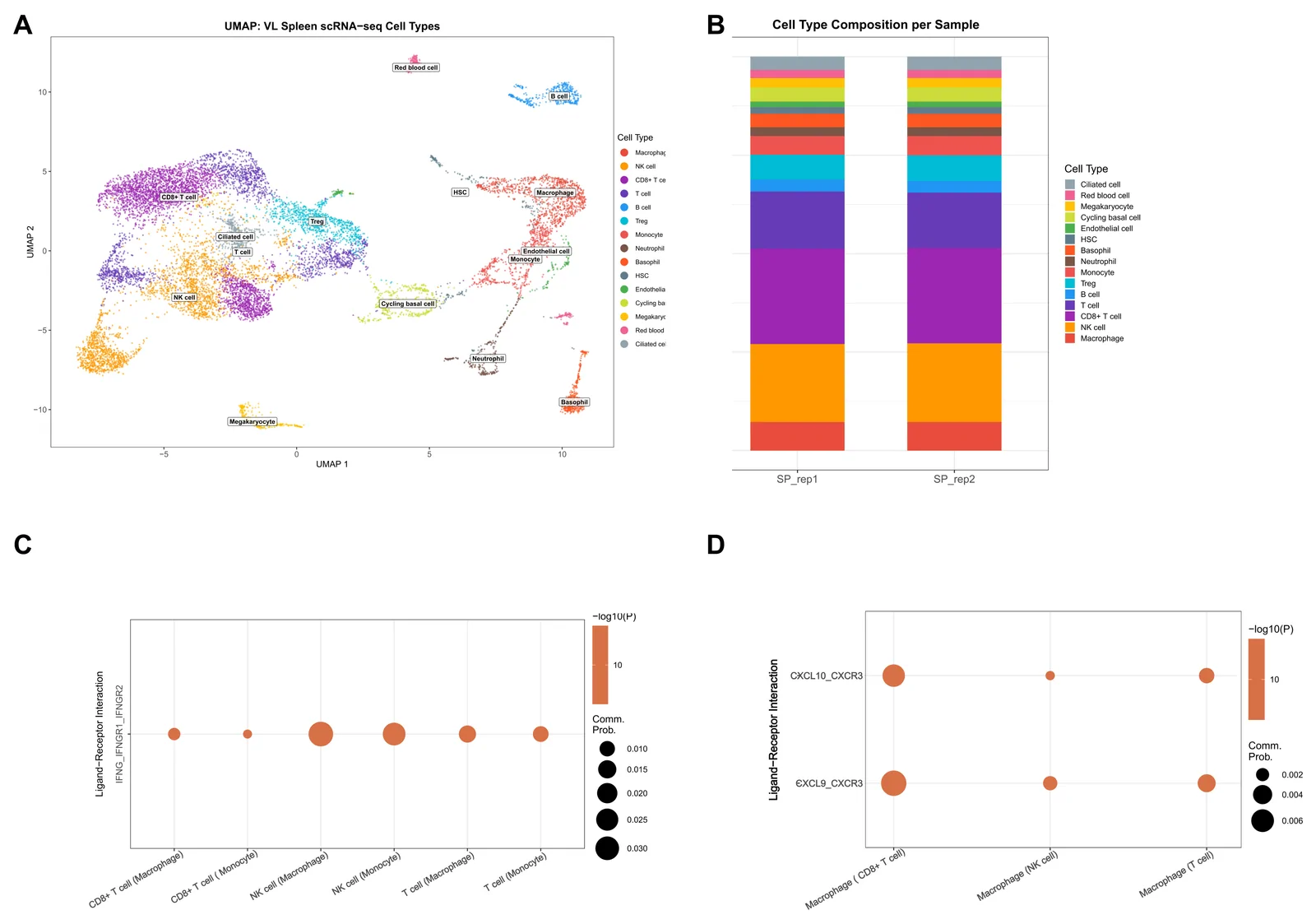
Supportive mouse spleen single-cell context and inferred CellChat communication. **(A)** UMAP of 15 cell types in two Leishmania-infected mouse spleen samples from GSE240385. **(B)** Cell-type composition by sample. **(C)** CellChat-inferred IFNG-IFNGR1/IFNGR2 communication from CD8-positive T cells, NK cells, and T cells toward macrophages or monocytes. **(D)** CellChat-inferred CXCL9/CXCL10-CXCR3 communication from macrophages toward CD8-positive T cells, NK cells, and T cells. CellChat probabilities are transcript-based associations and do not establish cytokine secretion or causal signaling.

CellChat inferred IFNG-IFNGR1/IFNGR2 communication from CD8-positive T cells, NK cells, and T cells toward macrophage or monocyte populations (**Figure 8C**), and CXCL9/CXCL10-CXCR3 communication from macrophages toward CD8-positive T cells, NK cells, and T cells (**Figure 8D**). These transcript-derived communication probabilities are supportive associations and do not demonstrate cytokine secretion, receptor activation, or causal intercellular signaling.

In the all-21-sample HMOX1 sensitivity analysis, HMOX1 expression was not significantly associated with the HMOX1-excluded heme-metabolism score (rho = -0.229, two-sided P = 0.319, bootstrap 95% CI -0.668 to 0.243; **Supplementary Figure 2A**) or the HMOX1-excluded inflammatory-response score (rho = 0.369, P = 0.0999, 95% CI -0.092 to 0.747; **Supplementary Figure 2B**). After both variables were adjusted for the heme-metabolism score, the residual association was also not significant (rho = 0.294, P = 0.197, 95% CI -0.195 to 0.692; **Supplementary Figure 2C**). These results do not provide evidence that HMOX1 is independently associated with the inflammatory-response score after accounting for the heme-related transcriptional signal.

### Signature-excluded WGCNA identifies a score-associated co-expression module in active VL

To address direct score-gene circularity, WGCNA was rerun *de novo* in all 21 GSE125992 exp2 samples after excluding all 132 score genes before variability filtering and network construction. Among powers 1-20, none reached signed scale-free topology R² >= 0.80, so the prespecified small-sample fallback power of 16 was used (**Figure 9A**). The 5,000 highest-MAD non-signature genes were assigned to modules using a signed network (**Figure 9B**).

**Figure 9:**
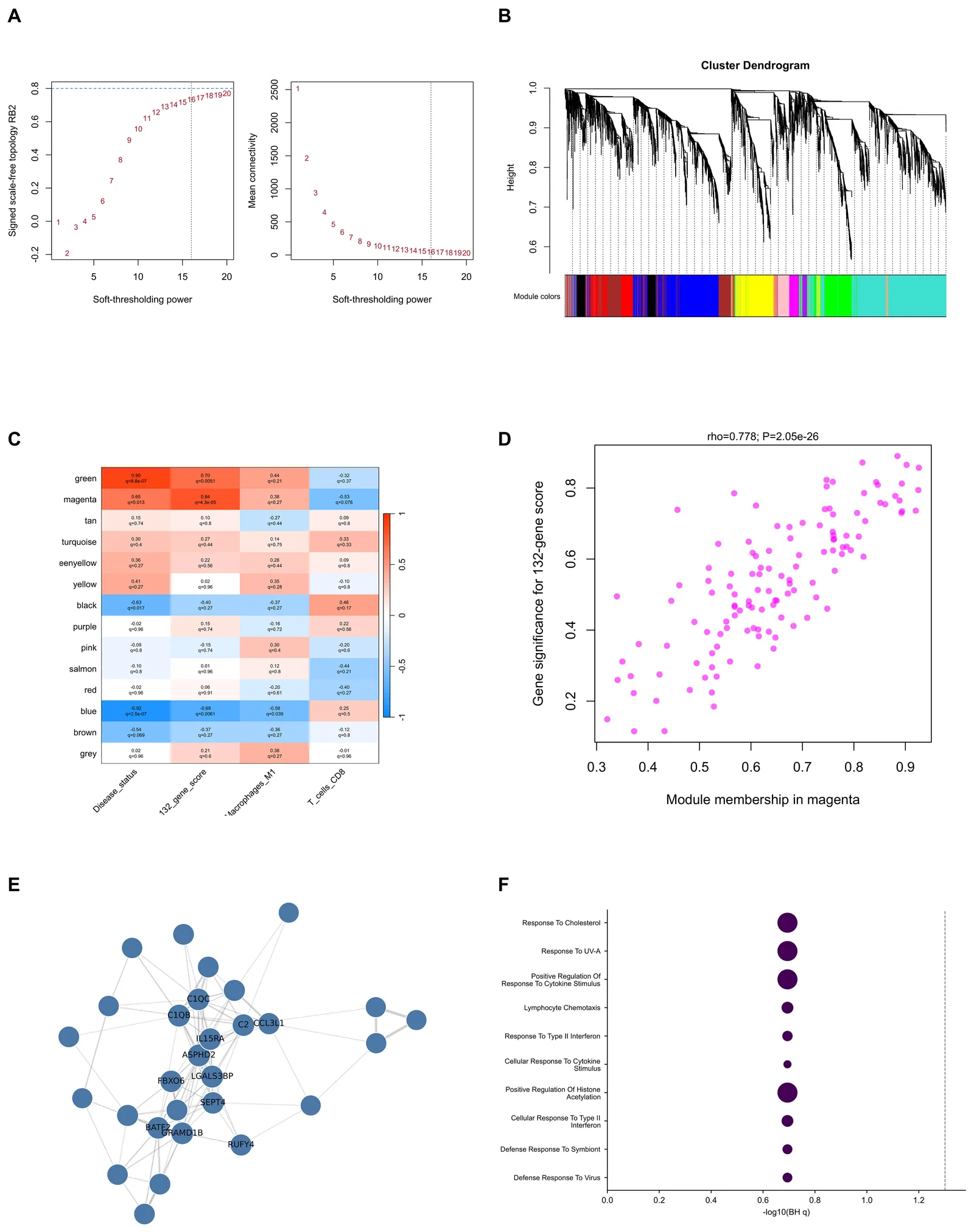
*De novo* signature-excluded WGCNA in GSE125992. All 132 exploratory score genes were excluded before variability filtering and network construction in all 21 exp2 samples. **(A)** Soft-threshold diagnostics for powers 1-20; none reached signed R² >= 0.80, so the prespecified small-sample fallback power 16 was used. **(B)** Dendrogram and modules from the 5,000 highest-MAD remaining genes. **(C)** Spearman module-trait correlations with BH correction across 56 tests. The 124-gene magenta module correlated with the exploratory score (rho = 0.837, raw P = 2.30 × 10^-6, BH q = 4.29 × 10^-5) and ActiveCase status (rho = 0.651, BH q = 0.0130), but not with M1 macrophages or CD8-positive T cells after correction. **(D)** Absolute module membership versus gene significance for the score (rho = 0.778, P = 2.05 × 10^-26). **(E)** Network of 30 high-membership magenta-module genes. **(F)** Exploratory GO-BP terms; no term reached BH q < 0.05 (minimum BH q = 0.202).

Fourteen modules were correlated with ActiveCase status, the exploratory 132-gene score, estimated M1 macrophage fractions, and estimated CD8-positive T-cell fractions, with BH correction across all 56 tests (**Figure 9C**). The 124-gene magenta module showed the strongest score association (rho = 0.837, raw P = 2.30 × 10^-6, BH q = 4.29 × 10^-5) and was also associated with ActiveCase status (rho = 0.651, BH q = 0.0130). Its associations with M1 macrophages (rho = 0.382, BH q = 0.275) and CD8-positive T cells (rho = -0.530, BH q = 0.0757) were not FDR-supported.

Within the magenta module, absolute module membership correlated with gene significance for the exploratory score (rho = 0.778, P = 2.05 × 10^-26; **Figure 9D**). A network of the 30 genes with the highest absolute module membership highlighted ASPHD2, LGALS3BP, C1QB, GRAMD1B, BATF2, IL15RA, C2, and C1QC among highly connected genes (**Figure 9E**). Because all 132 score genes were excluded before network construction, this association is not a direct consequence of shared gene membership.

Exploratory GO-BP analysis of the magenta-module genes included response to type II interferon, cytokine-response, and host-defense terms, but no term reached BH q < 0.05 (minimum BH q = 0.202; **Figure 9F**). The signature-excluded network therefore supports a correlated transcriptional module, not pathway-level FDR significance or causal mechanism.

### Development and same-study validation of an IFNG/GBP5/MX2 active-VL inflammatory classifier

The fixed GSE125992 analysis selected lambda.min = 0.02878664 and retained nonzero coefficients for IFNG, GBP5, and MX2 (**Figures 10A, B**). The frozen equation was logit(p) = -18.7752806 + 0.7503904 x IFNG + 2.2059416 x GBP5 - 1.1916386 x MX2. Training predicted probabilities differed between ActiveCase and EHC samples (Wilcoxon P = 5.67e-06, **Figure 10C**), and the apparent training AUC was 1.000 (bootstrap 95% CI 1.000-1.000, **Figure 10D**).

**Figure 10:**
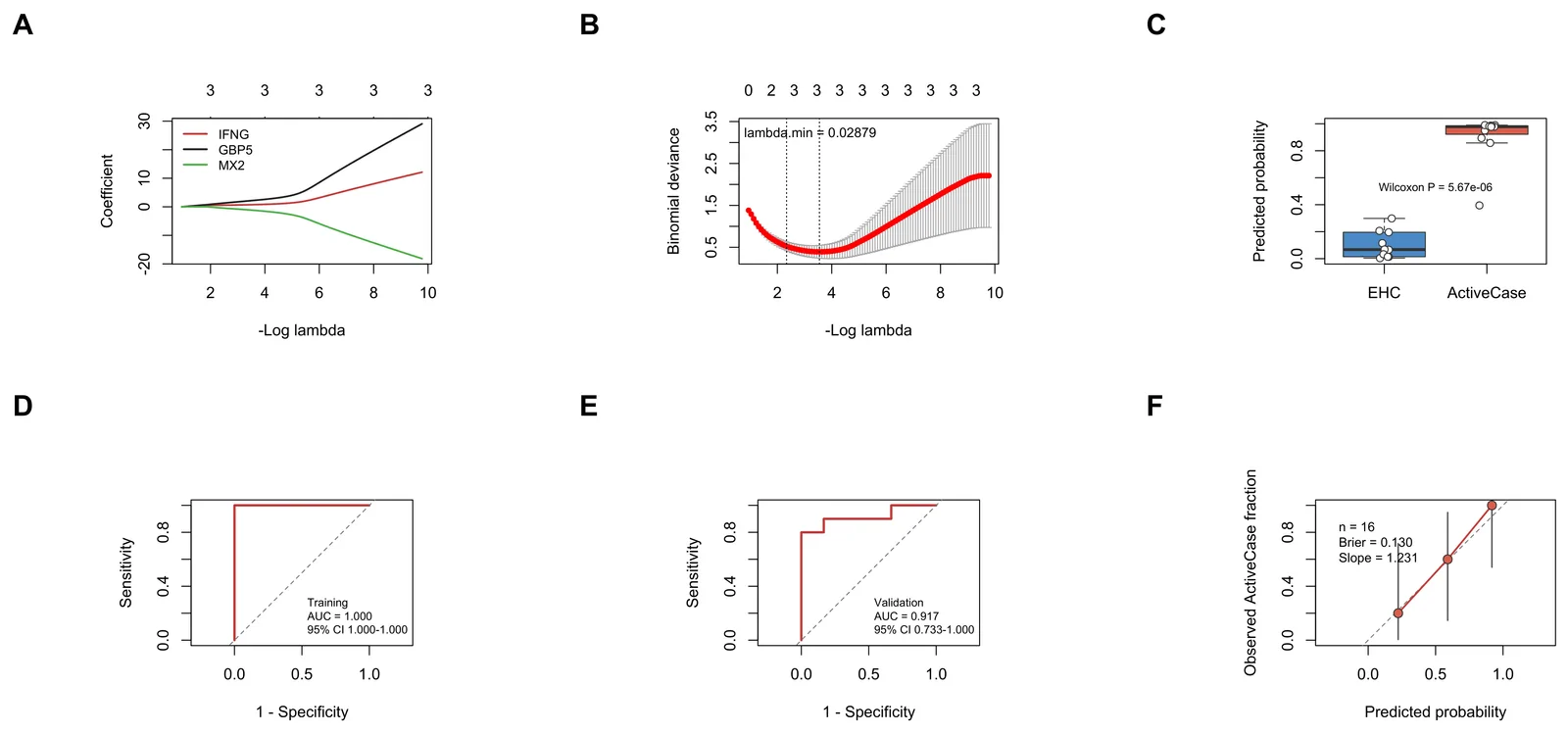
Development, same-study experiment-level validation, and exploratory calibration of the IFNG/GBP5/MX2 active-VL inflammatory classifier. **(A)** LASSO coefficient trajectories. **(B)** Fixed-fold ten-fold cross-validation selected lambda.min = 0.02878664 and retained nonzero coefficients for IFNG, GBP5, and MX2. **(C)** GSE125992 training probabilities (Wilcoxon P = 5.67 × 10^-6). **(D)** Training ROC curve (AUC = 1.000; bootstrap 95% CI 1.000-1.000). **(E)** Same-study validation in GSE125991 (AUC = 0.917; bootstrap 95% CI 0.733-1.000; sensitivity = 0.900 and specificity = 0.833 at p = 0.5). **(F)** Exploratory GSE125991 calibration (Brier score = 0.130; calibration slope = 1.231). The 16-sample validation cohort was not an independent external cohort.

The newly refitted and fully specified model was frozen before application to GSE125991, with no validation-set feature selection, coefficient refitting, or threshold optimization. In GSE125991, validation AUC was 0.917 (bootstrap 95% CI 0.733-1.000); at the prespecified threshold of 0.5, sensitivity was 0.900 and specificity was 0.833 (**Figure 10E**). Exploratory calibration yielded a Brier score of 0.130 and a calibration slope of 1.231 (**Figure 10F**), with wide bootstrap uncertainty because the validation cohort contained only 16 samples. GSE125991 and GSE125992 are experiments within the same SuperSeries, and participant-level overlap cannot be excluded; this is same-study experiment-level validation rather than independent external validation and does not establish clinical HLH prediction.

## Discussion

The active-VL whole-blood dataset showed IFN-associated, chemokine, antigen-presentation, and myeloid-related transcriptional changes, consistent with previous VL transcriptomic studies (Gardinassi et al., 2016; Fakiola et al., 2019; da Matta et al., 2023). CXCL9 and CXCL10 were higher after transcriptome-wide correction, whereas the STAT1 difference was more modest. These observations support an IFN-gamma-associated inflammatory state in active VL, but the public data do not demonstrate an IFN-gamma-driven causal loop.

The sorted CD68-positive monocyte comparison between pediatric HLH and severe sepsis provided an exploratory comparator rather than a matched validation cohort. The 132-gene score was derived from a nominal-P-selected contrast and was higher in active VL, but it may capture a broader IFN-associated inflammatory response rather than HLH-specific biology. Cross-platform projection by ssGSEA reduces the need for direct data merging but does not remove age, compartment, cellular-composition, platform, or disease-context confounding.

The IFN-gamma/STAT1/CXCL9-CXCL10 axis is therefore interpreted as a candidate association network in VL and an exploratory point of convergence with pediatric HLH (Griffin et al., 2020; Cron et al., 2023; Lin et al., 2021). The gene-expression, score, deconvolution, WGCNA, and CellChat findings are mutually contextualizing but remain observational and computational.

FARDEEP-LM22 identified FDR support for the M1 macrophage-score association but not for CD8-positive T-cell or resting or activated NK-cell associations. Excluding all 132 score genes before *de novo* WGCNA removed direct gene-membership circularity; the resulting magenta module remained associated with the score, although shared upstream biology and cross-sectional confounding remain possible. CellChat and correlation analyses cannot demonstrate cytokine secretion, pathway activation, or causal signaling.

HMOX1 expression may reflect erythrophagocytosis, heme load, hemolytic or oxidative stress, macrophage activation, or a mixture of these processes (Ryter et al., 2006). In the all-21-sample sensitivity analysis, HMOX1 was not significantly associated with the HMOX1-excluded heme-metabolism score (rho = -0.229, P = 0.319) or inflammatory-response score (rho = 0.369, P = 0.0999), and the heme-score-adjusted residual association was also not significant (rho = 0.294, P = 0.197; **Supplementary Figures 2A–C**). Thus, these transcriptomic data do not distinguish hemolysis or erythroid stress from myeloid inflammation and do not support an independent mechanistic attribution for HMOX1.

The clinical and demographic metadata available for the public cohorts were incomplete, particularly for comparator-specific age, sex, comorbidities, disease severity, and treatment covariates. This precluded participant-level subgroup stratification and multivariable adjustment for potential confounders. In addition, the analyses used fixed public cohorts, so a prospective power calculation was not applicable; the small cohort sizes and incomplete covariate data limit statistical precision and generalizability.

In summary, public transcriptomic integration supports an IFN-gamma-associated inflammatory state in active VL and exploratory cross-compartment convergence with pediatric HLH monocytes. The exploratory 132-gene score, M1 macrophage association, signature-excluded co-expression module, and same-study classifier validation are hypothesis-generating. Prospective cohorts with clinical HLH phenotyping and independent molecular validation are required before claims of HLH specificity, mechanism, diagnosis, prognosis, or clinical utility.

## Data Availability

The datasets presented in this study are publicly available in the NCBI Gene Expression Omnibus (GEO) repository under accession numbers GSE125992, GSE125991, GSE150707, and GSE240385.

## Supplementary material

Supplementary Figure 1 Expression patterns of selected IFN-associated inflammatory genes across cell populations in Leishmania-infected mouse spleen samples. **(A)** UMAP FeaturePlots showing Cxcl9, Cxcl10, Gbp2, Isg15, and Stat1 expression across quality-controlled cells from two Leishmania-infected mouse spleen samples in GSE240385. Color indicates normalized expression. These plots show transcript distribution in UMAP space and do not provide spatial localization. **(B)** DotPlot summarizing average expression and the percentage of expressing cells across annotated cell populations.

Supplementary Figure 2 Sensitivity analysis of HMOX1, heme metabolism, and inflammatory-response scores. HMOX1 was excluded from both MSigDB Hallmark gene sets before ssGSEA. All 21 GSE125992 exp2 samples were used (ActiveCase n = 11; EHC n = 10). **(A)** HMOX1 expression versus HALLMARK_HEME_METABOLISM ssGSEA NES (rho = -0.229, two-sided P = 0.319, bootstrap 95% CI -0.668 to 0.243). **(B)** HMOX1 expression versus HALLMARK_INFLAMMATORY_RESPONSE ssGSEA NES (rho = 0.369, P = 0.0999, 95% CI -0.092 to 0.747). **(C)** Spearman correlation between HMOX1 and inflammatory-score residuals after separate adjustment for the heme-metabolism score (rho = 0.294, P = 0.197, 95% CI -0.195 to 0.692). Confidence intervals used 2,000 sample-level bootstrap resamples.
