# Supplementary material for: Transcriptomic evidence of an IFN-gamma-associated inflammatory state in active visceral leishmaniasis: an exploratory comparison with pediatric HLH
Source: Front Cell Infect Microbiol. 2026 Oct 1;16:1924856. doi: 10.3389/fcimb.2026.1924856 (PMC13668529; doi:10.3389/fcimb.2026.1924856)
Supplement: Supplementary file 1 [file Table1.docx]

**Supplementary Table S1. GEO dataset eligibility criteria and analytical roles**

| **Dataset** | **Disease relevance** | **Comparator / sample basis** | **Data and QC availability** | **Objective eligibility basis** | **Decision / analytical role** | **Key limitation** |
| --- | --- | --- | --- | --- | --- | --- |
| GSE125992 | Human active VL and endemic controls | exp2 ActiveCase n = 11 vs EHC n = 10 for all GSE125992 analyses | Raw IDAT and processed matrix available; source study reports background correction, quantile normalization, log2 transformation and probe QC | Human whole-blood cohort with an explicit endemic-control group, adequate group labels, accessible expression data and direct relevance to VL discovery | Include: all 21 eligible exp2 samples for VL expression and enrichment, score construction, FARDEEP-LM22, signature-excluded WGCNA, and classifier training | Whole-blood cellular composition; incomplete participant-level metadata |
| GSE125991 | Human active VL and endemic controls | exp1 ActiveCase n=10 vs EHC n=6 | Raw IDAT and processed matrix available; same platform and source-study QC workflow as GSE125992 | Compatible platform, explicit case/control labels, and official processed expression values supported direct application of the refitted frozen model | Include: same-study experiment-level validation of the refitted frozen classifier without validation-set feature selection, coefficient refitting, or threshold optimization | Same GSE125993 SuperSeries as GSE125992; participant-level independence cannot be established from public metadata |
| GSE150707 | Pediatric active HLH contrasted with severe sepsis | Sorted CD68-positive monocytes: HLH n=12 vs severe sepsis n=8 | CEL/CHP and processed SST-RMA data available; sample labels specify disease and cell subset | Direct pediatric HLH disease context, clinically relevant inflammatory comparator and cell-enriched bulk expression suitable for gene-level reanalysis | Include: pediatric HLH disease anchor and exploratory cross-cohort convergence | Different compartment and platform from VL; no genes passed the prespecified global BH and absolute log2FC thresholds |
| GSE240385 | Chronic L. donovani infection in mouse spleen and bone marrow | Four infected samples; spleen replicates n=2 used; no uninfected controls | Raw and processed 10x files available with tissue, age, sex and infection-stage annotations | Single-cell resolution permits cell-type contextualization of inferred inflammatory programs in infected mouse spleen samples | Include only as cross-species cellular contextualization; exclude from human validation and pooled inference | Mouse-only, n=2 spleen samples, no uninfected comparator and different tissue/disease stage |

**Note:** Eligibility required a public expression resource with usable processed or raw data, explicit sample annotations, and relevance to a prespecified analytical role. A dataset without an appropriate comparator was not used for case-control inference; cross-species data were not treated as human validation; and experiments from the same SuperSeries were not treated as independent external cohorts. GEO, Gene Expression Omnibus; HLH, hemophagocytic lymphohistiocytosis; VL, visceral leishmaniasis.

**Supplementary Table S2. Normality, homoscedasticity,
 effect-size, and statistical-test selection details**

**A. Gene-expression comparisons**

| **Figure** | **Dataset; comparison (n1/n2)** | **Variable** | **Shapiro P (group 1/group 2)** | **Levene P** | **Assumption-guided sensitivity test** | **Primary analysis** | **Mean difference (bootstrap 95% CI)** | **Raw P** | **BH q** |
| --- | --- | --- | --- | --- | --- | --- | --- | --- | --- |
| Figure 5A | GSE125992; ActiveCase - EHC (11/10) | *STAT1* | 0.5346/0.9313 | 0.1175 | Student t-test (P=0.0337) | limma eBayes, trend=TRUE, robust=TRUE | 0.290 (0.049, 0.537) | 0.0298 | 0.1163 |
| Figure 5B | GSE125992; ActiveCase - EHC (11/10) | *CXCL9* | 0.5854/0.8209 | 0.0169 | Welch t-test (P=0.0006) | limma eBayes, trend=TRUE, robust=TRUE | 1.504 (0.899, 2.160) | 6.08e-5 | 0.0013 |
| Figure 5C | GSE125992; ActiveCase - EHC (11/10) | *CXCL10* | 0.3756/0.7776 | 0.4486 | Student t-test (P=0.0005) | limma eBayes, trend=TRUE, robust=TRUE | 1.957 (1.095, 2.828) | 0.000139 | 0.0024 |
| Figure 5D | GSE150707; HLH - Sepsis (12/8) | *STAT1* | 0.0844/0.1830 | 0.4405 | Student t-test (P=0.0097) | limma eBayes, trend=TRUE, robust=TRUE | 3.148 (1.291, 4.961) | 0.0060 | 0.8843 |
| Figure 5E | GSE150707; HLH - Sepsis (12/8) | *CXCL9* | 0.0040/0.5235 | 0.0679 | Wilcoxon rank-sum (P=0.1049) | limma eBayes, trend=TRUE, robust=TRUE | 3.039 (0.774, 5.733) | 0.0558 | 0.9998 |
| Figure 5F | GSE150707; HLH - Sepsis (12/8) | *CXCL10* | 0.0582/0.0023 | 0.0074 | Wilcoxon rank-sum (P=0.0372) | limma eBayes, trend=TRUE, robust=TRUE | 3.842 (1.167, 6.845) | 0.0343 | 0.9998 |

**Note:** Shapiro-Wilk and median-centered Levene tests are reported as sample-level assumption checks. The primary Figure 5A-F inference remained the prespecified limma empirical-Bayes model (trend=TRUE, robust=TRUE) with transcriptome-wide Benjamini-Hochberg correction; Student, Welch, or Wilcoxon results are sensitivity checks and did not replace the limma model. Mean differences are group 1 minus group 2 in log2-expression units, with 2,000-resample percentile bootstrap confidence intervals.

**B. FARDEEP-LM22 group comparisons**

| **Figure** | **Dataset; comparison (n1/n2)** | **Variable** | **Shapiro P (group 1/group 2)** | **Levene P** | **Assumption-guided sensitivity test** | **Primary analysis** | **Mean difference (bootstrap 95% CI)** | **Raw P** | **BH q** |
| --- | --- | --- | --- | --- | --- | --- | --- | --- | --- |
| Figure 7A | GSE125992 exp2; ActiveCase - EHC (11/10) | Monocytes | 0.7587/0.3327 | 0.8142 | Student t-test (P=0.0044) | Student t-test | -0.128 (-0.199, -0.051) | 0.0044 | 0.0171 |
| Figure 7A | GSE125992 exp2; ActiveCase - EHC (11/10) | Macrophages M1 | 0.0935/1.0000 | 0.0028 | Welch t-test (P=0.0085) | Welch t-test | 0.004 (0.002, 0.006) | 0.0085 | 0.0171 |
| Figure 7A | GSE125992 exp2; ActiveCase - EHC (11/10) | T cells CD8 | 0.8462/0.2417 | 0.1259 | Student t-test (P=0.1884) | Student t-test | -0.037 (-0.087, 0.013) | 0.1884 | 0.1884 |
| Figure 7A | GSE125992 exp2; ActiveCase - EHC (11/10) | NK cells activated | 0.0142/1.01e-5 | 0.0314 | Wilcoxon rank-sum (P=0.0466) | Wilcoxon rank-sum | 0.029 (0.007, 0.054) | 0.0466 | 0.0621 |

**Note:** For Figure 7A, the two-sided test was selected as follows: Wilcoxon rank-sum when either group had Shapiro-Wilk P<=0.05; Welch t-test when both groups had Shapiro-Wilk P>0.05 but Levene P<=0.05; otherwise Student t-test. BH q values control the four displayed group comparisons. Mean differences are ActiveCase minus EHC relative fractions, with 2,000-resample percentile bootstrap confidence intervals. These group comparisons are descriptive and are separate from the 22-cell correlation family in Supplementary Table S3.

**Supplementary Table S3. LM22 cell-fraction correlations with the exploratory 132-gene score in GSE125992**

| **Immune-cell type** | **n** | **Spearman rho** | **Raw P** | **BH q** | **Bootstrap 95% CI** | **Estimable** | **FDR-supported** |
| --- | --- | --- | --- | --- | --- | --- | --- |
| Macrophages M1 | 21 | 0.787 | 2.29e-5 | 0.000503 | 0.626, 0.847 | Yes | Yes |
| Plasma cells | 21 | 0.571 | 0.0069 | 0.0756 | 0.160, 0.863 | Yes | No |
| T cells gamma delta | 21 | 0.450 | 0.0405 | 0.2227 | 0.090, 0.714 | Yes | No |
| Macrophages M0 | 21 | 0.419 | 0.0586 | 0.2580 | -0.001, 0.755 | Yes | No |
| T cells CD4 memory activated | 21 | 0.393 | 0.0783 | 0.2735 | -0.046, 0.694 | Yes | No |
| NK cells activated | 21 | 0.297 | 0.1908 | 0.4663 | -0.178, 0.659 | Yes | No |
| T cells CD4 naive | 21 | 0.201 | 0.3815 | 0.7364 | -0.308, 0.614 | Yes | No |
| B cells naive | 21 | 0.123 | 0.5953 | 0.9354 | -0.333, 0.566 | Yes | No |
| B cells memory | 21 | 0.092 | 0.6925 | 0.9558 | -0.298, 0.489 | Yes | No |
| NK cells resting | 21 | 0.091 | 0.6951 | 0.9558 | -0.366, 0.530 | Yes | No |
| T cells follicular helper | 21 | 0.076 | 0.7419 | 0.9601 | -0.165, 0.320 | Yes | No |
| Mast cells activated | 21 | 0.013 | 0.9545 | 1.0000 | -0.457, 0.511 | Yes | No |
| Mast cells resting | 21 | NE | 1.0000 | 1.0000 | NE | No | No |
| Dendritic cells resting | 21 | NE | 1.0000 | 1.0000 | NE | No | No |
| Dendritic cells activated | 21 | -0.005 | 0.9814 | 1.0000 | -0.452, 0.426 | Yes | No |
| T cells regulatory (Tregs) | 21 | -0.012 | 0.9599 | 1.0000 | -0.485, 0.403 | Yes | No |
| Eosinophils | 21 | -0.173 | 0.4535 | 0.7675 | -0.481, 0.173 | Yes | No |
| Macrophages M2 | 21 | -0.193 | 0.4017 | 0.7364 | -0.605, 0.256 | Yes | No |
| T cells CD8 | 21 | -0.278 | 0.2225 | 0.4896 | -0.651, 0.186 | Yes | No |
| T cells CD4 memory resting | 21 | -0.369 | 0.0995 | 0.2735 | -0.684, -0.370 | Yes | No |
| Neutrophils | 21 | -0.377 | 0.0924 | 0.2735 | -0.650, 0.014 | Yes | No |
| Monocytes | 21 | -0.453 | 0.0391 | 0.2227 | -0.809, 0.037 | Yes | No |

**Note:** Spearman correlations used all 21 GSE125992 exp2 samples (ActiveCase n=11; EHC n=10) and the separate rank-normalized ssGSEA score reconstructed from 132 of 212 exploratory GSE150707 HLH-upregulated genes that mapped to GSE125992. BH correction covered all 22 LM22 cell types; confidence intervals used 2,000 bootstrap resamples. Resting mast cells and resting dendritic cells were constant at zero, so rho and confidence intervals were not estimable; both were conservatively retained as P=1 and q=1. BH, Benjamini-Hochberg; CI, confidence interval; EHC, endemic healthy control; FDR, false discovery rate; HLH, hemophagocytic lymphohistiocytosis; LM22, leukocyte signature matrix 22; NE, not estimable; ssGSEA, single-sample gene set enrichment analysis.
